# History and prospects of flax genetic markers

**DOI:** 10.3389/fpls.2024.1495069

**Published:** 2025-01-15

**Authors:** Daiana A. Zhernova, Elena N. Pushkova, Tatiana A. Rozhmina, Elena V. Borkhert, Alexander A. Arkhipov, Elizaveta A. Sigova, Ekaterina M. Dvorianinova, Alexey A. Dmitriev, Nataliya V. Melnikova

**Affiliations:** ^1^ Engelhardt Institute of Molecular Biology, Russian Academy of Sciences, Moscow, Russia; ^2^ Federal Research Center for Bast Fiber Crops, Torzhok, Russia; ^3^ I.M. Sechenov First Moscow State Medical University, Moscow, Russia; ^4^ Moscow Institute of Physics and Technology, Moscow, Russia

**Keywords:** flax, *Linum usitatissimum* L., DNA polymorphisms, DNA markers, GWAS, QTL, QTN, marker-assisted selection

## Abstract

Flax (*Linum usitatissimum* L.) is known as a dual-purpose crop, producing both fiber and oil, which have a wide range of uses. Successful flax breeding requires knowledge on the genetic determinants of flax traits. The former identification of molecular markers for valuable traits used labor-intensive and sometimes poorly reproducible approaches. However, they allowed an assessment of the genetic diversity of flax and its relatives, the construction of linkage maps, and the identification of some markers for important characteristics. The sequencing of flax whole genome triggered the development of genome-wide association studies (GWAS) and quantitative trait locus (QTL) mapping. QTLs and quantitative trait nucleotides (QTNs) were identified for valuable seed- and fiber-related features and for resistance to biotic and abiotic stressors. Cost-effective and accurate analysis of large number of genotypes for multiple markers simultaneously using microarrays or targeted deep sequencing became available, as well as HRM, TaqMan, KASP, and other fluorescence-based high-throughput methods for detecting DNA polymorphisms. However, most DNA markers identified in flax are ambiguously linked to trait expression and are not universally applicable. A major challenge remains the lack of knowledge on functional polymorphisms. To date, only a few are known, mainly mutations in the *FAD3* genes responsible for reduced linolenic acid content in linseed oil. For the further development of marker-assisted and genomic selection of flax, it is necessary to analyze exhaustively phenotyped sample sets, to identify DNA polymorphisms that determine valuable traits, and to develop efficient DNA test systems.

## 1 Introduction

Flax (*Linum usitatissimum* L.) is one of the world’s ancient crops (family Linaceae DC. ex Perleb), known since ancient Egypt and Mesopotamia (Saha and Hazra, 2004), although it was domesticated only 8,000-10,000 years ago. Flax is a dicotyledonous self-pollinated annual herbaceous plant (diploid chromosome set 2n = 2x = 30, genome size ~ 450 Mb (Green et al., 2008; Soto-Cerda et al., 2013; Dvorianinova et al., 2022; You et al., 2023). Flax is known as a dual-purpose crop, producing both fiber and oil. *L. usitatissimum* is one of the three most important textile crops in the world and one of the five most important oilseed crops (Deng et al., 2011; Ottai et al., 2011). Flax seed is a rich source of ω-3 (linolenic) and ω-6 (linoleic) fatty acids, easily digestible dietary fiber, and lignans, which have beneficial effects on the human body. In particular, they prevent and reduce the risk of cardiovascular diseases, various types of cancer, diabetes, atherosclerosis, arthritis, osteoporosis, autoimmune diseases, reduce cholesterol levels, stimulate blood flow, have a beneficial effect on the nervous system, and have antioxidant, anti-inflammatory, and antimicrobial effects (Kezimana et al., 2018; Campos et al., 2019; Parikh et al., 2019; Saini et al., 2021; Al-Madhagy et al., 2023). In addition, flax seeds are widely used in pharmaceuticals, animal feed, and for the production of environmentally friendly paints, varnishes, lubricants, linoleum, biofuels, etc (Singh et al., 2011; Corino et al., 2014; Goyal et al., 2014; Campos et al., 2019). Another valuable product derived from flax is fiber, which is used in the textile industry and for the production of paper and a variety of composite materials (Jhala and Hall, 2010; Fombuena et al., 2019; Asyraf et al., 2022).

The aim of our review is to describe the history of development of flax DNA markers and to propose the promising directions of their future in flax breeding for creation of varieties with a complex of valuable traits.

## 2 Development and application of molecular markers in flax

The beginning of the new millennium was characterized by a great interest in the use of molecular markers for plant studies. The first studies on flax used mainly dominant (Random Amplified Polymorphic DNA – RAPD, Amplified Fragment Length Polymorphism – AFLP, Inter Simple Sequence Repeat – ISSR, Inter-Retrotransposon Amplified Polymorphism – IRAP, Retrotransposon Microsatellite Amplified Polymorphism – REMAP, Sequence-Specific Amplified Polymorphism – SSAP, and Inter-Primer Binding Site – iPBS) and codominant (Restriction Fragment Length Polymorphism – RFLP and microsatellites or Simple Sequence Repeats – SSRs) markers.

RFLP markers were used in studies of flax varieties and hybrids to identify molecular markers for valuable traits and to characterize genetic diversity (Schneeberger and Cullis, 1991; Lawrence et al., 1993; Oh et al., 2000).

RAPD method was applied in studies of flax varieties, hybrids, landraces, and related to *L. usitatissimum* species for genotyping, characterization of genetic diversity, determination of phylogenetic relationships, and identification of molecular markers for valuable traits (Cullis et al., 1999; Stegniî et al., 2000; Bo et al., 2002; Fu et al., 2002a, 2002b, 2003a, 2003b; Muravenko et al., 2003; Fu, 2005; Diederichsen and Fu, 2006; Bo et al., 2008; Muravenko et al., 2009; Singh et al., 2009; Bibi et al., 2015; Nagabhushanam et al., 2021).

AFLP approach also enabled genotyping flax varieties, analyzing their genetic diversity, assessing genetic relationships between genotypes, and identifying markers for valuable flax traits using mapping populations (Spielmeyer et al., 1998; Everaert et al., 2001; Van Treuren et al., 2001; Wakjira et al., 2005; Adugna et al., 2006; Chandrawati et al., 2014).

SSR markers were widely used in flax studies to genotype varieties, evaluate their genetic diversity, assess their phylogenetic relationships, create their genetic maps, and identify markers for valuable traits using mapping populations and accession collections (Ellegren, 2004; Rashid and Duguid, 2005; Roose‐Amsaleg et al., 2006; Cloutier et al., 2009; Fu and Peterson, 2010; Deng et al., 2011; Rachinskaya et al., 2011; Soto-Cerda et al., 2011; Cloutier et al., 2012; Soto-Cerda et al., 2012; Asgarinia et al., 2013; Soto-Cerda et al., 2013, 2014; Chandrawati et al., 2017b; Choudhary et al., 2017a, 2017; Sudarshan et al., 2017; Wu et al., 2017; Chandrawati et al., 2017a; Saha et al., 2019; Soto-Cerda et al., 2019; Singh et al., 2021; Saroha et al., 2022b; Chen and Liu, 2024).

ISSR markers were applied to characterize the genetic diversity and relationships among flax varieties and *L. usitatissimum* wild ancestor *Linum bienne* Mill. and to identify markers for valuable traits (Wiesnerova and Wiesner, 2004; Rajwade et al., 2010; Uysal et al., 2010; El Sayed et al., 2018; Kocak et al., 2023).

Markers based on the analysis of sequences derived from retrotransposons were also used in flax research. SSAP, IRAP, REMAP, and iPBS markers allowed evaluation of genetic diversity and genotyping of flax varieties, characterization of relationships between species of the genus *Linum*, and assessment of activity of LTR retrotransposons in flax plants exposed to stressors (Smýkal et al., 2011; Melnikova et al., 2014; Habibollahi et al., 2015; Abbasi Holasou et al., 2016; Lancíková and Žiarovská, 2020; Žiarovská et al., 2022).

The combination of different types of dominant and codominant markers was utilized in flax studies for linkage map development, genotyping, and evaluation of genetic diversity and relationships among varieties (Oh et al., 2000; Kumari et al., 2018; Mhiret and Heslop-Harrison, 2018; Osman et al., 2021).

The molecular markers described above have significant weaknesses: insufficient number in the genome, inapplicability for complex polygenic traits, poor reproducibility, and labor-intensive experiments. The period of active use of dominant and codominant markers was replaced by the era of Single Nucleotide Polymorphisms (SNPs), which became possible due to advances in DNA sequencing technologies. The phylogenetic relationships of species in the genus *Linum* were studied using sequencing of non-coding regions of chloroplast DNA, and some hypotheses on the origin of cultivated flax were confirmed (Fu and Allaby, 2010). The combined use of EST (expressed sequence tag)-SSR and SNP markers on a doubled haploid population allowed the development of a linkage map for flax and the localization of markers for seed traits on chromosomes (Cloutier et al., 2011). However, a new level of flax research was opened by the transition from sequencing single loci to sequencing whole genomes or large sets of genomic regions. The sequencing of about 44 thousand BAC clones of the variety CDC Bethune enabled the construction of a genome-wide physical map of flax and the characterization of its genome (Ragupathy et al., 2011). The further development of flax molecular markers was largely driven by next-generation sequencing (NGS) technologies. Sequencing of reduced representation libraries for eight flax varieties identified about 55 thousand SNPs that could be useful for flax research and breeding (Kumar et al., 2012).

## 3 GWAS and QTL mapping in flax

Quantitative Trait Locus (QTL) mapping and Genome-Wide Association Study (GWAS) allow the identification of genomic regions responsible for valuable traits. QTL mapping uses biparental populations, while GWAS is based on the analysis of unrelated individuals (Gupta et al., 2019). These approaches can greatly advance the identification of DNA markers for valuable traits and accelerate marker-assisted and genomic selection (Adlak et al., 2023; Veerendrakumar et al., 2024). In 2012, the whole genome of flax was sequenced and assembled (Wang et al., 2012), facilitating GWAS and QTL mapping in this crop. We describe the relevant studies below and summarized the data on the identified QTLs in **Supplementary Table 1**. **Supplementary Table 1** lists QTLs associated with seed-related traits, fiber-related traits, resistance to biotic and abiotic stressors, and some other valuable flax traits. QTL coordinates on the reference genome and references to the works in which the QTLs were identified are also listed.

In many studies, authors identified hundreds or thousands of QTLs associated with different flax traits. In **Supplementary Table 1**, we included only the most significant QTLs, if they were highlighted by the authors of the studies.

Seed-related traits, which are particularly important for linseed, were in the focus of a significant number of works. QTLs for seed oil content and composition, seed protein content, seed yield and weight, and days to maturity were identified using the recombinant inbred line (RIL) population (Kumar et al., 2015). QTLs for seed oil traits, seed yield, and days to maturity were revealed using genome sequencing of biparental mapping populations (You et al., 2018a). Yield-related QTLs (thousand seed weight, capsule number, number of branches, fatty acid content) were also determined using Specific-Locus Amplified Fragment Sequencing (SLAF-seq) of the flax core collection (Xie et al., 2017, 2018, 2019). In addition, QTLs for seed size and weight were revealed by GWAS of the flax core collection (Guo et al., 2019), and Quantitative Trait Nucleotides (QTNs) for seed weight were identified using multi-locus GWAS (Saroha et al., 2023). QTLs associated with fatty acid composition of oil and its content in flax seeds were identified by Genotyping-By-Sequencing (GBS) of the RIL population (Zhao et al., 2020). SNPs associated with seed weight, seed oil content, days to flowering, and plant branching were revealed by GBS of a set of linseed varieties (Singh et al., 2019). Furthermore, QTLs associated with flowering time (Soto-Cerda et al., 2021) and mucilage and hull content (Soto-Cerda et al., 2018) and QTNs associated with flowering and maturity time (Saroha et al., 2022a) were identified by GWAS analysis of the flax core collections. In addition, SNPs associated with male sterility (Zhao et al., 2023) and QTLs for petal color (Guo et al., 2024) were revealed in flax using GWAS analysis.

The search for QTLs for fiber-related traits is another major direction in flax research, which is particularly important for fiber flax. QTLs for plant height, technical length, and fiber content were identified using SLAF-seq of the flax core collection (Xie et al., 2017, 2018). QTLs for plant height, technical length, straw weight, fiber content, and fiber yield were revealed using genome sequencing of biparental mapping populations (Wu et al., 2018; You et al., 2018a). QTLs for plant height and technical length were also identified using GBS of the RIL population (Zhang et al., 2018). SNPs associated with flax plant type (fiber flax or linseed) were revealed by the analysis of whole-genome sequencing data for the flax core collection (Povkhova et al., 2021). QTNs for fiber traits and plant height were revealed using GWAS (Kanapin et al., 2022; Saroha et al., 2022a).

In addition to traits related to seed and fiber, the resistance of flax plants to biotic and especially abiotic stressors is of interest. QTNs for drought resistance were identified by GWAS (Soto-Cerda et al., 2020; Sertse et al., 2021). QTLs for salt tolerance were also revealed (Li et al., 2022). QTNs for resistance to Fusarium wilt (*Fusarium oxysporum* f. sp. *lini*) were identified using GWAS (Kanapin et al., 2021) and RIL population analysis (Cloutier et al., 2024). QTLs for resistance to pasmo (*Septoria linicola*) were also revealed using GWAS (He et al., 2019, 2023). In addition, QTLs and QTNs for resistance to powdery mildew (*Oidium lini*) were identified by GWAS (Speck et al., 2022; You et al., 2022).

An important study to systematize the available data on flax QTLs and to determine their location on chromosomes of the variety CDC Bethune was published in 2020 (You and Cloutier, 2020). QTLs associated with flax resistance to biotic and abiotic stressors were summarized in the review of 2022 (Yadav et al., 2022). Fiber-related QTLs were also compiled in the study of 2024 (Gudi et al., 2024). Identification and systematization of flax QTLs are necessary to understand the genetic basis of flax traits and to effectively develop improved varieties using marker-assisted and genomic selection and genome editing. Currently, hundreds of flax genomes were sequenced, and sequencing data are available in databases. However, the lack of available and detailed descriptions of phenotypes of the sequenced samples is now becoming a more pressing problem. Such data would allow comprehensive analyses to identify the genetic determinants of valuable flax traits and to assess their diversity. In addition, almost all the studies described above used the genome assembly of the variety СDС Bethune (You et al., 2018b) as a reference. However, this genome was assembled without the use of long-read sequencing data and is less complete and accurate than flax genomes obtained later using third-generation sequencing platforms from Oxford Nanopore Technologies or Pacific Biosciences (Sa et al., 2021; Dvorianinova et al., 2022). Therefore, reanalysis of the genome sequencing data for flax samples using a more complete and accurate genome as a reference may improve the results of the QTL search. In addition, the construction of the flax pan-genome could also lead to the efficient localization of QTLs and QTNs. Furthermore, the identification of genes that play a role in the determination of valuable flax traits could be based on transcriptome data. Genes with high expression levels in specific tissues and developmental stages are likely to be involved in the processes occurring there and deserve attention for further research (Galinousky et al., 2020; Dvorianinova et al., 2023; Gorshkova et al., 2023).

## 4 Functional markers in flax breeding

Many QTLs were identified for valuable flax traits. However, QTLs for the same traits revealed in different studies often do not overlap, suggesting that the studied genotypes, the experimental design, and the data analysis have a significant influence on the obtained results. This hinders the widespread use of QTLs in flax breeding. However, some functional DNA markers, which are not only associated but specifically define valuable flax traits, were identified.

It was shown that nonsense and missense mutations in the *FAD3A* and *FAD3B* genes decrease linolenic acid and increase linoleic acid in linseed oil (Vrinten et al., 2005; Banik et al., 2011; Thambugala et al., 2013; Rajwade et al., 2016; Porokhovinova et al., 2019; Dmitriev et al., 2020). These markers are useful and are already used in linseed breeding to develop food varieties whose oil is more resistant to oxidation compared to traditional linseed varieties (Povkhova et al., 2022).

The researchers also revealed functional SNPs that determine the color of flax seeds. Mutations in the second exon of the *FLAVONOID 3′5′ HYDROXYLASE* (*F3′5′H*) gene negatively affect the synthesis of proanthocyanidins, resulting in a yellow seed coat (Sudarshan et al., 2017). Some of the SNPs in the glutathione S-transferase gene, which lead to four amino acid substitutions, are also likely to result in a yellow color of flax seed coats (Young et al., 2022). These markers are useful for flax breeding because yellow seeds may be more attractive for the use in food products (Abtahi and Mirlohi, 2024).

In addition, *S-lectin receptor-like kinase* (*SRLK*) (Lus10025891) was proposed as a candidate for a major flax resistance gene to Fusarium wilt. The SNP and indel, which resulted in amino acid substitutions in SRLK, distinguished resistant flax varieties from susceptible ones and probably determined the resistance (Cloutier et al., 2024). These markers could be used in the development of flax varieties with resistance to the harmful flax pathogen – *Fusarium oxysporum* f. sp. *lini*. Moreover, ethyl methanesulfonate induced a mutation in *LuALS1* that conferred resistance to sulfonylurea herbicides in flax plants (Liu et al., 2023).

## 5 Prospects of marker-assisted selection in flax

The use of functional DNA polymorphisms in plant breeding requires the utilization of simple and reliable assays. Cleaved Amplified Polymorphic Sequence (CAPS) markers were efficiently used in marker-assisted selection of crops (Shavrukov, 2014, 2016), including flax (Povkhova et al., 2022). However, less labor-intensive and more rapid methods are preferred for the analysis of large sample sets, and approaches based on fluorescence detection are promising. High-Resolution Melting (HRM) allows the identification of DNA polymorphisms based on the comparison of amplicon melting curves and is suitable for high-throughput genotyping of plants (Simko, 2016), including flax (Povkhova et al., 2022). HRM requires a DNA-intercalating dye, as opposed to fluorophore-labeled SNP-specific probes in the TaqMan method (Broccanello et al., 2018; Ayalew et al., 2019). TaqMan assays also allow for rapid and accurate genotyping of large sample sets (Woodward, 2014). Another method for fluorescence detection of DNA polymorphisms is Kompetitive Allele-Specific PCR (KASP) (He et al., 2014), which uses universal Fluorescence Resonance Energy Transfer (FRET) cassettes. FRET cassettes greatly simplify and/or cheapen genotyping, so KASP was used effectively for many crops (Dipta et al., 2024). The KASP markers were involved in QTL mapping and identification of the gene likely responsible for flax resistance to Fusarium wilt (Cloutier et al., 2024). Thus, KASP and similar approaches utilizing FRET cassettes are promising for the identification of SNPs and indels that define valuable flax traits, including in marker-assisted selection.

Microarrays were used extensively in recent years in large-scale studies where samples were needed to be analyzed for a large number of markers simultaneously. The technology allows DNA to be tested for the presence of target sequences by hybridizing to probes attached to a solid platform. This method is convenient for mass use when the same traits are analyzed in many samples, but it is disadvantageous for single use (Yang and Wei, 2015). Studies on flax using microarrays are rare (Roach and Deyholos, 2007; 2008; Fenart et al., 2010).

Targeted amplicon sequencing allows the complete sequence of a gene or locus of interest to be obtained and SNPs/indels to be analyzed (Senapathy et al., 2010; Cronn et al., 2012; Akhmetshina et al., 2020). This approach requires target enrichment, for instance by amplification or hybridization. Targeted sequencing expands horizons in areas such as plant breeding, genetics, evolutionary and phylogenetic studies (Mertes et al., 2011; Cronn et al., 2012; Andermann et al., 2020).

**Figure 1:**
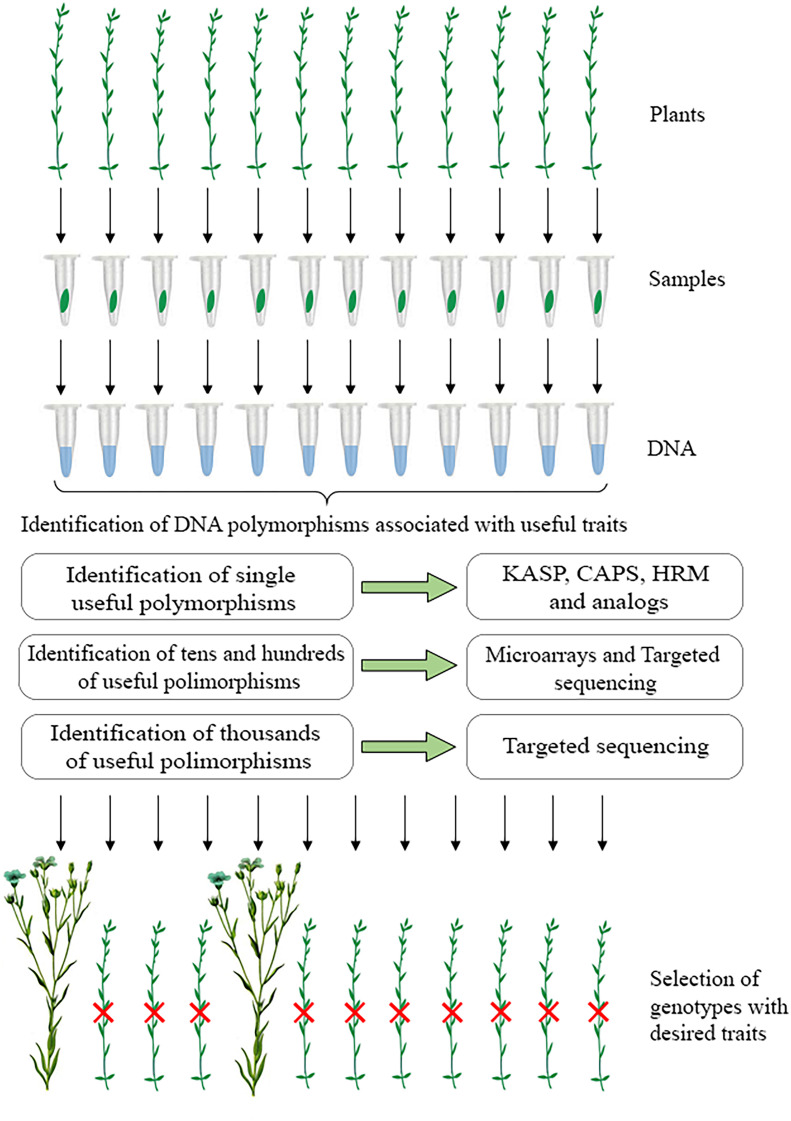
A scheme for marker-assisted selection in flax: collection of material from individual plants, isolation of DNA, testing samples for the presence of target DNA polymorphisms using various approaches, and selection of promising plants for breeding based on the obtained results.

The above methods can be applied in flax breeding. A scheme for marker-assisted selection in flax, in which different approaches can be used to identify nucleotides at sites of interest, is shown in **Figure 1**. It involves collecting material from individual plants, isolating DNA, testing samples for the presence of target DNA polymorphisms, and selecting promising plants for breeding based on the obtained results. The use of these technologies will increase the efficiency of creating improved flax varieties. At the same time, marker-assisted selection in flax requires the identification of a larger number of functional polymorphisms that determine the desired traits. This can be facilitated by extensive phenotyping of those flax samples for which genome sequencing data are available. As a result, rapid and accurate determination of flax plant traits based on effective DNA test systems will take flax breeding to the next level.
